# Evaluation of dry feeding and liquid feeding to lactating sows under high temperature environment

**DOI:** 10.1186/s40781-016-0118-0

**Published:** 2016-10-21

**Authors:** J. S. Hong, S. S. Jin, S. W. Jung, L. H. Fang, Y. Y. Kim

**Affiliations:** School of Agricultural Biotechnology, Seoul National University, Seoul, Korea

**Keywords:** Dry feeding, Lactating sow, Liquid feeding, Piglet, High temperature environment

## Abstract

**Background:**

Liquid feeding system has been introduced to domestic swine farms, but negative cognition about liquid feeding system has been remained for feed waste decay related with poor management and microbial contamination. For these reasons, this study was conducted to evaluate the effects of feeding method in lactating sows.

**Methods:**

A total of 30 mixed-parity (average 4.13) lactating sows (Yorkshire × Landrace) with an initial BW of 218.8 ± 19.5kg was used in a 3 week trial. Sows were allotted to 1 of 2 treatments in a completely randomized design by their body weight, backfat thickness, parity and alive litter weight. One of treatments was dry feeding and the other was liquid feeding (water to feed ratio, 1:1). Experimental diets contained 3265 kcal ME/kg, 12.6 % CP, 5.76 % EE, 1.09 % total lysine, 0.25 % total methionine, as fed basis.

**Results:**

Dry feeding treatment had high body weight loss rather than liquid feeding treatment (*P* = 0.04). Dry feeding treatment had tendency to increase litter weight at 21d of lactation (*P* = 0.06) and litter weight gain (*P* = 0.04) during lactation period (0–3 week). Sows fed dry feeding method made milk containing high content of casein and total solid rather than sows fed liquid feeding method (*P* = 0.04). In addition, dry feeding treatment had tendency to higher content of milk fat, protein and solid not fat on 21d of lactation (*P* = 0.07). Sows fed dry feeding type also showed higher milk energy content in milk of 21d lactation (*P* = 0.05). Furthermore, liquid feeding treatment showed high occurrence in feed waste during lactation period (*P* < 0.01).

**Conclusion:**

Dry feeding method was more suitable feeding method to lactating sows under high temperature environment like lactating barn.

## Background

Since nutrient utilization of lactating sows was focused on milk production, insufficient nutrient intake caused body composition loss for maintain milk production [13]. Therefore low feed intake during lactation caused severe body weight loss, delayed WEI, reduced conception rate, ovulation rate and fetus survival rate [1, 5, 6, 22]. Some research had been demonstrated that liquid feeding group had higher feed intake rather than dry feeding group [7, 8, 14].

Generally lactating sows were accommodated in high temperature environment for suckling piglets in Korea. For these reasons, liquid feeding system has been introduced to domestic swine farms for improving feed efficiency and feed intake under high temperature environment. However, negative cognition about liquid feeding system has been remained for feed waste decay related with poor management and microbial contamination. Therefore this study was conducted to evaluate the effect of dry feeding and liquid feeding on the productivity and feed intake of lactating sows.

## Methods

### Animal and housing

All experimental procedures involving animals were conducted in accordance with the Animal Experimental Guidelines provided by the Seoul National University Institutional Animal Use and Care Committee (SNUIAUCC; SNU-160613-13).

A total of 30 gestating sows (Yorkshire × Landrace, average parity 4.13) with an initial BW of 218.8 ± 19.5 kg were used in a 3 week lactation experiment. Sows were allotted to 1 of 2 treatments in a completely randomized design by their body weight, backfat thickness, parity and litter weight. When day 110 of gestation, sows were moved into individual farrowing crates (2.5 × 1.8 m^2^) and housed until weaning. Before 5 days of parturition day, each experimental diet was provided to sows for adaptation with reducing the diet 200g/d until predicted day of parturition. After parturition, they fed the diet increased gradually from 1kg/d by 1 kg/d until 5 day postpartum (5kg at 5day postpartum). After 5 day postpartum, feed and water were provided *ad libitum* to sows. Lactating sows were provided a feeder and a waterer, separately. Within 24 h postpartum, Fe-dextran (150ppm) injection, ear notching, needle teeth clipping and tail docking were practiced to all piglets. Piglets were cross-fostered across treatments within 1 day after birth to balance suckling intensity of sows with equalization of litter size, and thus to minimize any impact of initial litter size potentially affecting litter growth. In addition, male piglets were castrated in 3 days postpartum. During lactation period, all lactating sows and their progeny were raised in individual farrowing barn where the indoor temperature was regulated by automatic ventilation system and room temperature was kept automatically at 27–30 °C by heating lamps.

### Experimental design and diet

Treatment was composed of dry feeding and liquid feeding. Dry feeding treatment was only fed the solid feed. Liquid feeding treatment was fed the liquid-form diet with mixed solid feed and water (1:1). Feeding time was at 08:00, 11:00, 14:00, 17:00, 20:00, 23:00 and once feeding amount was ad libitum until 1 h after feeding time. In next feeding time, we checked the status of feed in feeder for determining the feed waste or not. Feed was used commercial lactating sows’ diet produced by commercial company in Korea. The chemical composition of experimental feed was presented in Table 1.

**Table 1:** Chemical composition of experimental feed

Chemical composition	%
Metabolizable energy, kcal ^a^	3265.00
Ether extract ^b^, %	5.76
Crude protein ^b^, %	12.60
Total lysine ^a^, %	1.09
Total methionine ^a^, %	0.25
Crude ash ^b^, %	4.95
Total calcium ^b^, %	0.45
Total phosphorus ^b^, %	0.50

^a^ Calculated value

^b^ Analyzed value

### Measurements

Body weight and backfat thickness of sows were measured at 24 h postpartum and 21 day of lactation. Body weight of sows was measured by weight machine (CAS, Korea). An ultra-sound device (Renco lean meter, Renco Corp., Minneapolis, USA) was used for measuring backfat thickness at P_2_ position (mean value from both side of the last rib and 65 mm away from the backbone). The piglet weight was recorded within 24 h postpartum and at 21 day of lactation and the number of piglets was recorded after cross-fostering and at 21 day of lactation. Litter weight was calculated by summing the individual piglet weights from one sow at after-fostering and 21d of lactation. Weaning to estrus interval was recorded from day of weaning to day of first estrus sign. Feed intake was recorded during experimental period (0–3 week) and feed waste was checked frequently when sows fed the dry-form and liquid-form diet. Feed waste was determined the status of feed in individual feeder, such as sour smell of feed and animal behavior (feed intake rejection). After the feed waste was taken out, feed waste was dried by drying oven and weighted. Colostrum and milk samples were taken from the 5 sows of each treatment at 24 h postpartum and 21 day lactation, respectively. After injection of oxytocin 0.3ml (10IU/ml) through ear vein, milks were collected from first or second teat of lactating sow. After milk collection in conical tube (50ml), samples were stored in a freezer (−20 °C) until further analysis. Proximate analysis of colostrum and milk (21d) was conducted using Milkoscan FT 120 (FOSS Electric). The milk production (g), milk dry matter (g) and milk energy (kcal) were calculated from piglet weights and growth rates by equations in Noblet and Etienne [11]. Milk production (g) = 2.50 × piglet ADG (g) + 80.2 × initial piglet BW (kg) + 7, Milk DM (g) = 0.401 × piglet ADG (g) + 12.9 × initial piglet BW (kg) + 19, Milk energy (kcal) = 2.54 × piglet ADG (g) + 78.7 × initial piglet BW (kg) + 153. Also, the energy content of milk at colostrum and 21d of lactation was calculated by equation of Perrin [17] through milk composition such as fat, protein, lactose. Milk energy (cal/100gl) = 9.11 fat(%) + 5.86 protein(%) + 3.95 lactose(%).

### Statistical analysis

The experimental data was analyzed using Student’s *t*-test procedure of SAS (SAS Inst., Inc., Cary, NC), and a main effect in the statistical model was lactation feeding method. For analyzing sow performance, litter performance and other collected data, individual sow and each litter were considered as an experimental unit. Probability values less than 0.05 (*P* < 0.05) were considered as significant difference; *P* < 0.10 were indicative of a trend; and values equal to or greater than 0.10 were considered as non-significant difference.

## Results

Dry feeding treatment had greater body weight loss than liquid feeding treatment (Table 2, *P* = 0.04). However, there was no significant difference in backfat thickness and its changes. Daily feed intake had no difference among treatment and weaning to estrus interval (WEI) also was not affected by feeding type.

**Table 2:** Effect of dry feeding and liquid feeding on physiological status in lactating sows ^a^

Criteria	Treatment ^b^		SEM ^c^	*P*-value
	Dry feeding	Liquid feeding		
Body weight, kg				
24 h postpartum	224.03	213.73	3.561	0.15
Day 21 of lactation	215.73	217.07	3.016	0.83
BW changes (0-21d)	−8.30	3.34	2.816	0.04
Backfat thickness, mm				
24 h postpartum	18.9	20.6	1.07	0.44
Day 21 of lactation	18.2	20.1	0.90	0.29
BF changes (0-21d)	−0.7	−0.5	0.68	0.87
Daily feed intake, kg/d	6.16	5.87	0.183	0.43
WEI ^d^, d	4.36	4.50	0.128	0.28

^a^ A total of 30 lactating sows (Yorkshire x Landrace) with an initial BW of 218.8 ± 19.5 kg were used in a 3 week trial

^b^ Dry feeding: solid feed only, liquid feeding: solid feed 50%: water 50% mixed

^c^ Standard error of mean

^d^ WEI: weaning to estrus interval

The number of piglets at after cross-fostering and 21d of lactation had no significant difference among treatment (Table 3). Dry feeding treatment had a tendency to increase litter weight at 21d of lactation rather than liquid feeding treatment (*P* = 0.06). Moreover, litter weight gain during lactation period (0–3 week) had greater in sows fed dry feed type (*P* = 0.04). Nevertheless the result of litter weight, piglet weight (3 week) and piglet weight gain (0–3 week) did not show any significant difference.

**Table 3:** Effect of dry feeding and liquid feeding on litter performance in lactating sows

Criteria	Treatment		SEM ^a^	*P*-value
	Dry feeding	Liquid feeding		
No. of piglets				
After cross-fostering	13.00	12.93	0.242	0.89
Day 21 of lactation	11.73	11.40	0.196	0.40
Litter weight, kg				
After cross-fostering	19.41	19.29	0.571	0.92
Day 21 of lactation	64.63	57.16	1.985	0.06
Weight gain (0-21d)	45.22	37.87	1.781	0.04
Piglet weight, kg				
After cross-fostering	1.50	1.49	0.040	0.84
Day 21 of lactation	5.51	5.05	0.161	0.16
Weight gain (0-21d)	4.00	3.56	0.153	0.15

^a^ Standard error of mean

Milk dry matter, energy content and production amount during lactation period (0–3 week) was not affected by dry feeding or liquid feeding (Table 4). As a result, sows fed dry feeding type showed higher milk energy content in milk of 21d lactation (*P* = 0.05).

**Table 4:** Effect of dry feeding and liquid feeding on milk production in lactating sows

Criteria	Treatment		SEM ^a^	*P*-value
	Dry feeding	Liquid feeding		
Milk production ^b^				
Milk dry matter, g	114.81	106.25	3.002	0.16
Milk production, g	603.98	550.60	18.715	0.16
Milk energy content				
Milk (0-21d) ^b^, kcal/g	755.35	701.15	18.987	0.16
Colostrum ^c^, cal/100g	137.77	151.94	8.009	0.41
Milk (21d) ^c^, cal/100g	121.02	104.74	4.303	0.05

^a^ Standard error of mean

^b^ Equations from Noblet and Etienne [11]

- Milk production (g) = 2.50 × piglet ADG (g) + 80.2 × initial piglet BW (kg) + 7

- Milk DM (g) = 0.401 × piglet ADG (g) + 12.9 × initial piglet BW (kg) + 19

- Milk energy (kcal) = 2.54 × piglet ADG (g) + 78.7 × initial piglet BW (kg) + 153

^c^ Equations from Perrin [17]

- Milk energy (cal/100gl) = 9.11 fat(%) + 5.86 protein(%) + 3.95 lactose(%)

Milk composition of colostrum had no difference between dry feeding and liquid feeding excluding lactose (Table 5). Sows fed dry feeding method showed higher lactose content of colostrum than sows fed liquid feeding method (*P* = 0.04). In otherwise, sows fed dry feeding method made milk containing high content of casein and total solid rather than sows fed liquid feeding method (*P* = 0.04). In addition, dry feeding treatment had tendency to higher content of milk fat, protein and solid not fat on 21d of lactation (*P* = 0.07).

**Table 5:** Effect of dry feeding and liquid feeding on milk composition of colostrum and milk at 21d lactation

Criteria	Treatment		SEM ^a^	*P*-value
	Dry feeding	Liquid feeding		
Milk composition				
Casein, %				
24 h postpartum	6.03	6.52	0.256	0.37
Day 21 of lactation	4.76	4.18	0.153	0.04
Fat, %				
24 h postpartum	8.29	9.57	0.845	0.48
Day 21 of lactation	7.20	5.81	0.384	0.07
Protein, %				
24 h postpartum	7.51	8.36	0.350	0.25
Day 21 of lactation	5.34	4.66	0.194	0.07
Lactose, %				
24 h postpartum	4.61	4.01	0.140	0.04
Day 21 of lactation	6.11	6.21	0.042	0.28
Total solid, %				
24 h postpartum	22.50	24.36	0.997	0.38
Day 21 of lactation	20.09	18.05	0.534	0.04
Solid not fat, %				
24 h postpartum	12.52	12.72	0.333	0.78
Day 21 of lactation	11.50	10.94	0.158	0.07
Free fatty acid, %				
24 h postpartum	3.02	3.09	0.143	0.81
Day 21 of lactation	8.98	8.72	0.646	0.85

^a^ Standard error of mean

In current study, liquid feeding treatment showed high occurrence in feed waste during lactation period (Table 6, *P* < 0.01). Furthermore, feed waste resulted from liquid feeding treatment caused severe economic losses (Table 6, *P* < 0.01).

**Table 6:** Effect of dry feeding and liquid feeding on economic loss in swine farm

Criteria	Treatment		SEM ^a^	*P*-value
	Dry feeding	Liquid feeding		
Daily feed intake, kg/d	6.16	5.87	0.183	0.43
Total feed wastage, kg	0.75	7.97	0.985	<0.01
Feed waste cost ^b^, won	376	3986	492.6	<0.01

^a^ Standard error of mean

^b^ Feed cost: 500 won/kg

## Discussion

The result of body weight loss during lactation (0 to 3 week) was in accordance with Peng et al. [15]. They presented that sows fed with wet feeding had greater feed intake and less body weight loss rather than those of dry feeding during lactation. In addition, O’Grady and Lynch [14], Koketsu [7], and Lynch [8] demonstrated that lactating sows had higher feed intake 12, 11, and 7 %, respectively when the feed was wet or watery. However, the result of body weight loss in drying feeding treatment was caused from not daily feed intake but growth of suckling piglets. Because lactating sows usually meet their nutrient requirement for milk production primarily from feed intake but also from body protein and fat tissue storage [9]. Moreover, milk production of lactating sow is dependent upon the litter [2, 19]. Thus high litter weight gain during lactation demonstrated high milk production of lactating sows [11]. When energy intake is often not sufficient to support milk production, and sows will mobilize body lipid and protein stores to support lactation [10, 12]. Therefore increasing milk production or litter weight gain caused to greater body weight loss of lactating sows under same dietary feed or nutrient intake [10].

The occurrence of feed waste in liquid feeding treatment caused to different result of average daily feed intake during lactation compared with previous studies [7, 8, 14, 15]. In the research of Peng et al. [15], wet feeding treatment was fed self-automatically (wet/dry system with nipple drinker inside the feeder). On the contrary to this, current study prepared the liquid feed just before the feeding and fed some amount of liquid feed continuously until end of feed intake. For this experimental environment, feed waste of liquid feeding treatment occurred inevitably same with Peng et al. [15]. Under high temperature environment like lactating barn (27–30 °C), microbial contaminated wet feed waste or liquid type of feed waste which often occurred because of highly microbial activity [4]. It led to rejection of feed intake in lactating sows and negative effects on milk production and milk quality. The nutritional importance of mammary amino acid uptake did not match the amino acid profile in the milk [3] and the amino acid requirements during lactation would be higher than current estimates. For instance, Richert et al. [18] demonstrated a response in litter weight gain and dietary valine level and Pettigrew [16] reported that lysine requirement increased linearly as daily litter growth rate increased during lactation. Unfortunately, microbial contamination within liquid feed wastage broke down the amino acids balance of dietary feed [20] and reduced dietary energy content of feed [21]. It led to negative influence on milk production and milk quality. Since sows were not provided fresh liquid feed and sanitary feeder environment was always with same amount of daily feed intake, sows fed dry feeding had high milk component(casein, fat protein, total solid, solid not fat) and high energy content at milk of 21d lactation.

## Conclusion

Lactating sows fed dry feeding had greater body weight loss compared with liquid feeding group. It led to high content and quality of milk production and good litter performance during lactation period. Considering the feed waste under high temperature environment and economic loss, dry feeding method was more suitable feeding method to lactating sows under high temperature environment like lactating barn.

## Abbreviations

ADG: Average daily gain
BW: Body weight
CP: Crude protein
DM: Dry matter
EE: Ether extract
ME: Metabolizable energy
NRC: National Research Council
WEI: Weaning to estrus interval
